# The impact of occupational stress on nurses’ caring behaviors and their health related quality of life

**DOI:** 10.1186/s12912-016-0178-y

**Published:** 2016-09-27

**Authors:** Pavlos Sarafis, Eirini Rousaki, Andreas Tsounis, Maria Malliarou, Liana Lahana, Panagiotis Bamidis, Dimitris Niakas, Evridiki Papastavrou

**Affiliations:** 1 Department of Nursing, Cyprus University of Technology, Limassol, Cyprus; 2 Hellenic Open University, Faculty of Social Sciences, Patra, 26335 Greece; 3 Centers for the Prevention of Addictions and Promoting Psychosocial Health of Municipality of Thessaloniki, Thessaloniki, 54634 Greece; 4 Aristotle University of Thessaloniki, Medical School, Thessaloniki, 54124 Greece

**Keywords:** Occupational stress, Nurses, Health-related quality of life, Caring behaviors

## Abstract

**Background:**

Nursing is perceived as a strenuous job. Although past research has documented that stress influences nurses’ health in association with quality of life, the relation between stress and caring behaviors remains relatively unexamined, especially in the Greek working environment, where it is the first time that this specific issue is being studied. The aim was to investigate and explore the correlation amidst occupational stress, caring behaviors and their quality of life in association to health.

**Methods:**

A correlational study of nurses (*N* = 246) who worked at public and private units was conducted in 2013 in Greece. The variables were operationalized using three research instruments: (1) the Expanded Nursing Stress Scale (ENSS), (2) the Health Survey SF-12 and (3) the Caring Behaviors Inventory (CBI). Univariate and multivariate analyses were performed.

**Results:**

Contact with death, patients and their families, conflicts with supervisors and uncertainty about the therapeutic effect caused significantly higher stress among participants. A significant negative correlation was observed amidst total stress and the four dimensions of CBI. Certain stress factors were significant and independent predictors of each CBI dimension. Conflicts with co-workers was revealed as an independent predicting factor for affirmation of human presence, professional knowledge and skills and patient respectfulness dimensions, conflicts with doctors for respect for patient, while conflicts with supervisors and uncertainty concerning treatment dimensions were an independent predictor for positive connectedness. Finally, discrimination stress factor was revealed as an independent predictor of quality of life related to physical health, while stress resulting from conflicts with supervisors was independently associated with mental health.

**Conclusion:**

Occupational stress affects nurses’ health-related quality of life negatively, while it can also be considered as an influence on patient outcomes.

## Background

Occupational stress can be defined as a situation wherein job-related factors interact with an employee, changing his/her psychological and physiological condition in a way that the person is forced to deviate from normal functioning [1].

Work-related stress can be damaging to a person’s physical and mental health, while its’ high levels have been related/connected to high staff truancy and low levels of productivity. According to the American Institute of Stress, stress is a major factor in up to 80 % of all work-related injuries and 40 % of workplace turnovers [2].

Nursing is perceived as a strenuous job with high and complicated demands. The high job demands and the combination of too much responsibility and too little authority have been identified as some of the primary sources of occupational stress amid nursing staff [3–7].

Occupational stress may affect significantly nurse’s quality of life, and simultaneously reduce the quality of care. Caring is an interpersonal procedure defined by expert nursing, interpersonal sensitivity and intimate relationships, including positive communication and implementation of professional knowledge and skills [8]. Job related stress has as a result loss of compassion for patients and increased incidences of practice errors and therefore is unfavorably associated to quality of care [9]. Numerous studies show that it has a direct or indirect impact on the delivery of care and on patient results [10–12].

### Purpose of the study

The main purpose of this study is to investigate the relation between nurses’ working stress and the patient care behaviors as well as nurses’ health-related quality of life. According to the main hypotheses, occupational stress leads to the deterioration of nurse’s physical and mental health status, while it is negatively affecting the adoption of good practices concerning nurses’ caring behaviors.

## Method

### Study design-sample

A correlational study was conducted. In total, 300 questionnaires were distributed to nurses working in one public General Hospital and 3 private ones. The final sample consisted of 246 nurses (higher education graduates) and nursing assistants (high school or post-secondary education) (Response Rate 82 %). The composition of the sample reflects the Greek reality. Greece has the third lowest density of nurses (3.3 per 1. 000 population) in OECD countries after Turkey and Mexico, while many working positions in health-care units are covered by nursing assistants than Registered nurses [13]. Inclusion criteria for nurses were as follows: willingness to partake in, at least 1 year of work experience, with immediate association with patients.

### Research instruments

#### Socio-demographics

The first part of the questionnaire contained questions recording socio-demographic and work-related characteristics of the sample.

#### Caring behaviors

The Greek Version of the Caring Behaviors Inventory scale (CBI-GR) was used [14]. There are 4 correlated dimensions within its 24 items: (1) Assurance of Human Presence - items 16,17,18,20, 21, 22, 23 and 24 (2) Professional Knowledge and Skills - items 9,10,11,12 and 15 (3) Patient Respectfulness - items 1, 3, 5, 6, 13 and 19) Positive Connectedness – items 2, 4, 7, 8 and 14. Each item is ranked on a 6-point Likert scale from 1 = never to 6 = always. The higher the score, the more the nurse expresses the specific caring behavior. Total and subscale scores can be derived from the instrument. Papastavrou et al., [14] translated, adapted and cross-validated the 24-item English Version of CBI into Greek and evaluated its’ psychometric properties. The CBI-GR was proved to be comparable with the original 24-item English Version and suitable to measure nurse caring among Greek-speaking nurses [14].

#### Occupational stress

The Expanded Nursing Stress Scale (ENSS) for the investigation of nurses’ work related stress is one of the most widely used scales which has already been adapted and validated in Greek and developed by Gray-Toft & Anderson [15]. It incorporates 59 items with 9 subscales. Each item requires respondents to rate on a five-point Likert scale ranging from “1 never stressful” to “4 extremely stressful” and “0 does not apply”. The higher the score, the more agreeable the replier is to the situation being stressful. Total and subscale scores can be derived from the instrument. The subscales include: 1. limisted knowledge in dealing with death and dying 2. Conflicts with other employers 3. feeling unqualified to aid with the patient and their family emotional needs 4. Peer –related problems 5. conflicts with supervisor and accepting the least possible support by the charge nurse, immediate supervisor and administrators 6. workload 7. uncertainty concerning treatment and receiving insufficient information of their medical condition from physicians 8. fear to fail nursing tasks due to patients’ and their families’ irrational demands 9. feeling discriminated and isolated by nursing colleagues and other professionals. Adding all the scores from the 59 items we get the total stress score. [16]. ENSS demonstrated improved reliability (α = .96) [16] over the original NSS (α = .89) [15]. The translation and validation of the questionnaire was made by Moustaka et al., [17], who granted permission to use it.

#### Quality of life

SF-12, which measures physical and mental health status was used for the quality of life assessment. SF-12 includes 12 questions: 2 concerning physical functioning, 2 regarding role limitations caused by physical health problems, 1 question about bodily pain, 1 with reference to general health perceptions, 1 on vitality, 1 in regard to social functioning, 2 in relevance to role limitations because of emotional problems and 2 questions referring to general mental health [18]. It was constructed as a shorter alternative of the SF-36 Health Survey, which although it has proved to be useful for a variety of purposes, is too long for inclusion in some large-scale health measurement [18]. Translation and validation of the questionnaire was made by Kontodimopoulos et al. [18].

#### Ethical considerations

The Ethics Committees of both the public General Hospital and the private ones granted permission for conducting the research. The questionnaires were anonymous and self-administered. Nurses meeting the inclusion criteria were verbally requested to participate in the study. Each attendant was free to take part, refuse or withdraw at any time, without any consequences.

### Data analysis

Demographic data were analysed using descriptive statistics. Mean values (SD) were used to describe quantitative variables, which were portrayed as absolute and relative frequencies. The associations of two continuous variables were analysed by Pearson correlations coefficients. In order to investigate the association of stress factors with SF-12 summary scores and the CBI subscales multiple linear regression analysis was conducted after adjusting for sex, age, family status, having children, educational level, working sector, working experience, shift and working position. All reported *p* values are two-tailed. Statistical significance was set at *p* < 0.05 and analyses were conducted using SPSS statistical software (version 18.0).

## Results

### Respondent demographics

The sample consisted of 246 nurses with mean age 39.7 years (SD = 8.2 years). Sample characteristics are presented in Table 1. Most participants were women (85.8 %) and 63.4 % of them were married, while 39.6 % were technological institutions’ graduates. Most of the participants (54.9 %) were nursing assistants, as Greece is in the 32^nd^ place of out of 34 OECD countries rank at the number of nurses (per 1000 population).

**Table 1:** Sample characteristics

	*N* (%)
Sex	
Women	211 (85.8)
Men	35 (14.2)
Age (years), mean (SD)	39.7 (8.2)
Family status	
Unmarried	77 (31.3)
Married	156 (63.4)
Divorced	10 (4.1)
Widowed	3 (1.2)
Children	
Yes	158 (64.2)
No	88 (35.8)
Degree	
University	11 (4.5)
Technical university	97 (39.6)
2 year Technical School	78 (31.9)
High school	57 (23.3)
Other	2 (0.8)
Post-graduate degree	
No	236 (95.9)
Yes	10 (4.1)
PhD	
No	244 (99.2)
Yes	2 (0.8)
Working sector	
Public	218 (88.6)
Private	28 (11.4)
Total years in nursing, mean (SD)	15.3 (9.1)
Years in nursing in current job, mean (SD)	8.0 (7.0)
Shift	
Morning	58 (23.9)
Rotated	185 (76.1)
Working position	
Nursing assistant	135 (54.9)
Nurse	88 (35.8)
Supervisor of department	17 (6.9)
Supervisor of sector	5 (2.0)
Head of department	1 (0.4)

### Mean values of the ENSS, SF-12 and CBI scales

**Fig. 1:**
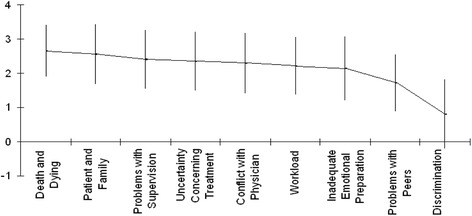
Mean values of ENSS subscales in order of importance

Mean values of study scales are provided in Table 2. More stressful factors were those that were related to death and dying (mean value m = 2.65; SD = 0.76) and those related with patient and family (m = 2.56; SD = 0.88) (Fig. 1).

**Table 2:** Mean values of study parameters

	Mean (SD)
ENSS	
Death and Dying Stressors	2.65 (0.76)
Patient and Family Stressors	2.56 (0.88)
Problems with Supervision Stressors	2.39 (0.86)
Uncertainty Concerning Treatment Stressors	2.34 (0.86)
Conflict with Physician Stressors	2.29 (0.88)
Workload Stressors	2.20 (0.83)
Inadequate Emotional Preparation Stressors	2.13 (0.94)
Problems with Peers Stressors	1.71 (0.84)
Discrimination Stressors	0.79 (1.01)
Total stress score	2.22 (0.65)
SF-12	
Physical component summary score	45.02 (7.63)
Mental component summary score	45.50 (11.18)
CBI	
Assurance of human presence	4.90 (0.76)
Professional knowledge and skill	5.07 (0.73)
Respect for patient	4.60 (0.84)
Positive connectedness	4.42 (0.89)

The least stressful factors were those that were related to discrimination (m = 0.79; SD = 1.01). Mean total stress was 2.22 (SD = 0.65). Also, mean value in physical component summary score was 45.02 (SD = 7.63) and in mental component summary score was 45.50 (SD = 11.18). Highest mean values in CBI scales were found in “Professional knowledge and skill” (m = 5.07; SD = 0.73) and in “Assurance of human presence” (m = 4.90; SD = 0.76).

### Occupational stress and its correlation with quality of life and caring behaviors

Correlation between ENSS scales and SF-12 and CBI scales are provided in Table 3. Mental component summary (MCS) score was negatively correlated with almost all ENSS scales except for “Discrimination Stressors”, indicating that more stressors are related with poorer mental health. Also, the physical component summary (PCS) score was significantly negatively correlated with stressors that had to do with discrimination, workload, problems with peers and supervision, indicating that more stressors in the afore-mentioned sectors are related with poorer physical health. Significantly negative correlations were found between almost all ENSS and CBI subscales. Thus, more stressful factors are related with worse behavior of the participants towards their patients.

**Table 3:** Correlation between stress scales and SF-12 and CBI scales

	SF-12		CBI			
ENSS	Physical component summary score	Mental component summary score	Assurance of human presence	Professional knowledge and skill	Respect for patient	Positive connectedness
Death and Dying Stressors	−0.03	−0.26***	−0.17**	−0.13*	−0.19**	−0.19**
Inadequate Emotional Preparation Stressors	−0.09	−0.25**	−0.25***	−0.22**	−0.21**	−0.16*
Discrimination Stressors	−0.13*	−0.07	−0.12	−0.15*	−0.17**	−0.11
Workload Stressors	−0.13*	−0.28***	−0.28***	−0.21**	−0.28***	−0.27***
Uncertainty Concerning Treatment Stressors	−0.07	−0.31***	−0.27***	−0.22**	−0.31***	−0.31***
Conflict with Physician Stressors	−0.08	−0.29***	−0.26***	−0.14*	−0.31***	−0.31***
Problems with Peers Stressors	−0.13*	−0.26***	−0.31***	−0.33***	−0.30***	−0.27***
Problems with Supervision Stressors	−0.14*	−0.32***	−0.27***	−0.18**	−0.31***	−0.34***
Patient and Family Stressors	−0.05	−0.33***	−0.21**	−0.04	−0.21**	−0.20**
Total stress score	−0.12	−0.35***	−0.31***	−0.23***	−0.33***	−0.33***

*p < 0.050 **p < 0.010 ***p < 0.001

Multiple regression results with SF-12 scales as dependent variables and stress subscales as independent, adjusted for demographics and other sample characteristics are given in Table 4.

**Table 4:** Multiple regression results with SF-12 scales as dependent variables and stress subscales as independent, adjusted for demographics and other sample characteristics

	Physical component summary score		Mental component summary score	
	β (SE)^a^	*P*	β (SE)^a^	*P*
Death and Dying Stressors	0.64 (0.70)	0.366	−2.45 (1.00)	0.015
Inadequate Emotional Preparation Stressors	−0.32 (0.54)	0.560	−2.45 (0.76)	0.002
Discrimination Stressors	−1.03 (0.49)	0.036	−0.75 (0.71)	0.294
Workload Stressors	−0.86 (0.63)	0.171	−3.09 (0.88)	0.001
Uncertainty Concerning Treatment Stressors	−0.33 (0.59)	0.577	−3.07 (0.82)	<0.001
Conflict with Physician Stressors	−0.28 (0.58)	0.625	−3.13 (0.80)	<0.001
Problems with Peers Stressors	−0.81 (0.60)	0.181	−3.27 (0.84)	<0.001
Problems with Supervision Stressors	−0.92 (0.60)	0.126	−3.38 (0.84)	<0.001
Patient and Family Stressors	0.17 (0.60)	0.778	−3.37 (0.84)	<0.001
Total stress score	−0.70 (0.81)	0.391	−4.98 (1.12)	<0.001

^a^regression coefficient (standard error) adjusted for sex, age, family status, having children, educational level, working sector, working experience, shift and working position

The desire for resigning was independently associated with physical health, with those who wanted to abandon the nursing profession confronting more physical symptoms. On the other hand, specialty, working sector and resignation desire, were independent predicting factors for quality of life related with mental health.

Only discrimination stressors were found to be negatively related to PCS scores while all other stressors were found to be negatively related to MCS scores. Also, after adjusting for demographics it was found that stress in total was negatively related to all CBI subscales. Additionally, stress regarding Workload, Uncertainty Concerning Treatment, Problems with Peers and Supervision were negatively related to all CBI subscales. Stress caused by Conflict with Physician was negatively related to “Assurance of human presence”, “Respect for patient” and “Positive connectedness”. Stress caused by Discrimination Stressors was negatively related to “Professional knowledge and skill” and “Respect for patient”.

Multiple regression results with CBI scales as dependent variables and stress subscales as independent, adjusted for demographics and other sample characteristics are given in Table 5.

**Table 5:** Multiple regression results with CBI scales as dependent variables and stress subscales as independent, adjusted for demographics and other sample characteristics

	Assurance of human presence		Professional knowledge and skill		Respect for patient		Positive connectedness	
	β (SE)^a^	*P*	β (SE)^a^	*P*	β (SE)^a^	*P*	β (SE)^a^	*P*
Death and Dying Stressors	−0.06 (0.07)	0.340	−0.08 (0.07)	0.250	−0.10 (0.07)	0.168	−0.11 (0.08)	0.185
Inadequate Emotional Preparation Stressors	−0.10 (0.05)	0.042	−0.10 (0.05)	0.051	−0.09 (0.06)	0.130	−0.06 (0.06)	0.306
Discrimination Stressors	−0.08 (0.05)	0.092	−0.10 (0.05)	0.024	−0.13 (0.05)	0.013	−0.08 (0.06)	0.149
Workload Stressors	−0.19 (0.06)	0.001	−0.15 (0.06)	0.008	−0.23 (0.07)	0.001	−0.25 (0.07)	<0.001
Uncertainty Concerning Treatment Stressors	−0.20 (0.05)	<0.001	−0.15 (0.05)	0.005	−0.26 (0.06)	<0.001	−0.28 (0.06)	<0.001
Conflict with Physician Stressors	−0.17 (0.05)	0.002	−0.08 (0.05)	0.121	−0.24 (0.06)	<0.001	−0.24 (0.06)	<0.001
Problems with Peers Stressors	−0.18 (0.06)	0.001	−0.23 (0.05)	<0.001	−0.21 (0.06)	0.001	−0.20 (0.07)	0.004
Problems with Supervision Stressors	−0.21 (0.06)	<0.001	−0.15 (0.06)	0.008	−0.29 (0.06)	<0.001	−0.31 (0.07)	<0.001
Patient and Family Stressors	−0.11 (0.06)	0.056	0.02 (0.06)	0.715	−0.13 (0.06)	0.042	−0.13 (0.07)	0.059
Total stress score	−0.27 (0.08)	<0.001	−0.21 (0.07)	0.006	−0.35 (0.08)	<0.001	−0.35 (0.09)	<0.001

^a^regression coefficient (standard error) adjusted for sex, age, family status, having children, educational level, working sector, working experience, shift and working position

The resignation desire was an independently predicting factor for all the CBI dimensions, with nurses who did not want to leave their job having higher scores in the “Assurance of human presence” and those who wanted to retire having lower score in the rest of the three remaining dimensions. Conflicts with co-workers was independent predicting factor for the “Assurance of human presence”, “Professional knowledge and skills” and “Respect for patient” dimensions, conflicts with doctors predicted the “Respect for patient” dimension and conflicts with supervisors was an independent predicting factor for the “Positive connectedness”. In all the above cases, the higher the stress on behalf of the conflicts was, the lower the score in the CBI dimensions was. Finally, the working sector was an independently predicting factor for the “Assurance of human presence” with nurses who worked in the private sector having higher scores.

## Discussion

The present study provided empirical support for the existence of stress experience in the nursing profession. The existence of anxiety symptoms among Greek nursing personnel complies with the findings from other researcher’s. In a study concerning the degree of anxiety and related symptoms in emergency nursing personnel in Greece, anxiety levels were found to be high among women and employees in public hospitals [19]. Moreover, the Nursing Stress Scale was used on 120 newly qualified nurses and 128 fourth-year student nurses in Ireland, to measure and compare the perceived levels of job-related stress and stressors. The perceived levels of stress were high in both groups. The topics that were concluded from the responses of both groups included extreem workload, strenuous working relationships and ill- provided clinical learning needs, while student nurses also reported the combination of academic demands with clinical placement [20].

All dimensions of CBI scales scored high, showing that participants tended to give answers to the positive part of the research tool, considering that high quality caring is the right of all patients and a responsibility of all nurses. The above trend highlights nurses’ perceptions about the importance of their acts and it is in agreement with literature review [21, 22].

The frequency of different caring behaviors reflects nurse’s perception about what caring is. The higher grade in caring behaviors was associated to the area of “Professional Knowledge and skills” which was followed by “Assurance of human presence” and “Patient respectfulness”. The above data is not in agreement with the findings of other studies in which psychosocial caring behaviors within the health care field were ranked as most important by nursing staff [23, 24].

Total stress was associated with the four dimensions of caring behaviors. Therefore, occupational stress may be considered as a predictor for the adoption of caring behaviors. High degrees of stress may add to suboptimal care, increased rates of safety breaches, and higher frequency in errors in everyday clinical practice [25–27]. In a previous study concerning work environment and nurse caring where CBI was used and 128 nurses participated, a statistically significant negative correlation between stress and nurse caring for the total CBI score and assurance, respectfulness and connectedness subscales was revealed [28].

The multivariate analysis revealed that “Problems with Peers Stressors” was an important and independent factor for the “Assurance of Human Presence”, which means that higher levels of perceived stress because of the conflicts with colleagues were related with lower levels of implementation of human presence behaviors. Meanwhile, “Problems with Peers Stressors” were independently correlated with “Professional knowledge and skills” dimension.

In addition, multivariate analysis showed a significant inverse association between both “Problems with Peers” and “Conflict with Physicians” stressors and “Patient respectfulness” behaviors. In other words behaviors that integrate respect for the patient are reduced by increase of conflict with peers’ and doctors’ levels.

Problems with peers and conflicts with doctors may lead nurses to spend a lot of energy coping with the difficulties that rose from these aspects, holding them at the same time away from focusing on patient needs. On the contrary, good interpersonal relationships in the workplace may contribute to the reinforcement of positive caring behaviors. In a study of Burtson & Stichler [28], satisfaction with coworkers subscale was positively correlated with the CBI total score. Caring and job satisfaction (including satisfaction with co-workers) were also positively correlated in a study where 1,091 medical-surgical staff nurses participated [29].

Results of many surveys indicated that when doctors and nurses had an effective cooperation, patients were more prone to report satisfaction with their care [30–32].

Patient satisfaction, which reflects patient’s perception of care received compared with the care expected, has been used as an indicator of quality of services provided by health care personnel [33, 34]. Patients bond more with nurses, since they take care of their day-to-day needs. They support patients and their families both physically and emotionally, while their key role is obviously more relational than technical [31, 32, 34, 35].

According to the multivariate analysis “Problems with Supervision” and “Uncertainty Concerning Treatment” stressors were important and independent factors for the “Positive Connectedness” dimension. This finding suggests that uncertainty about treatment and problems with supervisors may lead to the reduction of caring behaviors that include positive connectedness. Although past research has documented the importance of managerial support [36] and the importance of continuous education [37], for the decrease of job stress the impact and the administration of the specific stressors on the implementation of caring behaviors remains relatively unexamined.

In the current survey, the factor “Dealing with death and dying” caused in the participants higher stress, compared with all other stressors. The most stressful dimension was mainly related with coping with the reality of human suffering before the death-dying process, than the fact of the death itself. This complies with other research data with unmet expectations and feeling of regret about not being able to prevent an inevitable death may be a great source of stress, affecting nurse’s ability to function effectively [38, 39].

“Patients and their families” was the second most significant stressor. The continual interaction, the lack of cooperation and the nurse’s sense of feeling unprepared to cope with their different emotional needs create feelings of anger, fear and disappointment in nursing staff, leading to higher stress levels [40, 41].

Problems with patients and their families extend from absence of cooperation to violence behaviors. Literature review has revealed that the risk of physical and psychological violence on behalf of abusive patients and their relatives is a great stressor [42]. The experience in the USA is similar, revealing that workplace violence is a significant stressor, especially for Emergency Department nurses [43]. Verbal or physical abuse often had a negative psychological effect on nurses after the incident [44].

Problems with supervisors was the third most significant stressor, while multivariable analysis showed that it was independently correlated with nurses’ mental health. This could be attributed both to the lack of well-trained supervisors and to the existing “conflict with power” culture in Greece [44]. In Japan, less job control was associated with anxiety, while poorer supervisor support was most obviously associated with depression [45]. According to Health and Safety Executive in United Kingdom, lack of understanding and support from nursing head managers contributes significantly to work-related stress, [3] while greater supervisory support is associated with reduced stress and job satisfaction [46].

Concerning “Uncertainty about treatment”, the most stressing factor reflects the uncertainty about patient’s health and many times is due to doctors’ tendency to not sufficiently inform both nurses and patients [47]. Under these circumstances, the fact of confronting patients who endure various levels of distress and have different anticipations may guide nurses to the point being emotionally overwhelmed [48].

Finally according to the study findings, high levels of professional stress are strongly related to nurses’ perception of health related quality of life, which is something that has been reported by many researchers. In a recent Chinese study, occupational stressor (showed by role insufficiency and physical environment), personal strain (pointed out by physical and psychological strain), job burnout (exhibited by emotional exhaustion and professional efficacy) and duration of work hours, were proved to be among the main risk factors for nurses’ quality of life [49].

As far as physical health is concerned, the results confirm the literature evidence, in which work-related stress correlates with many physical health problems including migraines, muscle, back and joint pain [50], long term physical illnesses, hypertension [51], irritable bowel syndrome and duodenal ulcer [52] and immune and endocrine system illnesses [53].

The findings also suggest that occupational stress is associated with mental health problems. Many studies have reached similar findings [54, 55]. On emotional level, occupational stress has been correlated with anxiety, dysthymia, low self-esteem, depression and feelings of inadequacy, while in many cases it has been increasingly recognized as a major risk factor for mild psychiatric morbidity [19, 56, 57]. Multivariate analysis showed that specialty, working sector and resignation desire, were independent predicting factors related to mental health. Among nurses who had specialty, those working in the private sector had better mental health, which can be attributed, on the one hand, to the better organization of private units or the smaller range of duties. Finally, nurses considering retirement had worse mental health, which is consistent with the literature [58].

At the same time, excessive occupational stress has a negative consequence on the psychological well-being of hospital employees (including behavioral, emotional and cognitive levels), reducing their work efficiency. The study results are similarly observed by other researchers who reported that health professionals’ occupational stress is associated with low job satisfaction, negative work attitudes and negative consequences in the quality of health care providing [59–61].

### Limitations

The most important limitation of the study is the variability of nurses’ educational and professional levels, and specifically the large number of nurses’ assistants that were included in the sample. We assumed that nursing assistants may be most vulnerable to stress factors. Fewer professional qualifications may affect their emotional regulation, in contrast to those with higher educational level, that provides more specific training and skills. In addition, except for the inadequate training, lower educational level is correlated with less career prospects that affect mediating variables for occupational stress like work ability [62]. Moreover, the sample size was quite small, since the participants were selected on the basis of convenience, to which extent the study findings have limited generalizability.

## Conclusion

Findings suggest that nurses’ exposure to stress-related factors can be considered as a predictor of their caring behaviors implementation, while this also affects their health-related quality of life negatively. More specifically, conflicts with co-workers were independent predictors for assurance of human presence, professional knowledge and skills and patient respectfulness dimensions, conflicts with doctors for respect for patient dimension and conflicts with supervisors and uncertainty concerning treatment for positive connectedness dimension. As far as health related quality of life is concerned, discrimination stress factor was an independent predictor for physical health, while stress resulting from conflicts with supervisors was independently associated with mental health.

Study findings could help devise interventions that reduce, minimize or eradicate some of these stressors. Nurse’s ability to cope with the demands and stress from work may be improved with specific occupational health education and specific training programs that improve their knowledge and ability. Concerning to their content, primary (that that are related with stressors reducing), secondary (that target to individual response to stressors modification), and tertiary (that focus on specific assistance to those who experience high levels of stress) interventions must be implemented [63]. Concerning to their direction, they must be based both on individual and organizational level [63]. Workshops targeting to facilitation and verbalization of feelings, normalization of experience, relaxation techniques teaching, conflicts solving and positive reappraisal may help both to stress responses modification and stress coping [63–67]. Through the above tasks and procedures, on the one hand nurses could be taught ways to create positive meaning from difficult situations and on the other they may be helped to discover effective stress coping strategies in an individual level [65, 67]. In addition, interventions at an institutional and organizational level, including additional supervisor support, staff recognition policies, and more breaks provision, may be proved helpful to more supportive work environments establishment, preventing stress on a primary level [63–67].
